# Validation of ACR/EULAR definition of remission in rheumatoid arthritis from RA practice: the ESPOIR cohort

**DOI:** 10.1186/ar3896

**Published:** 2012-06-29

**Authors:** Bin Zhang, Bernard Combe, Nathalie Rincheval, David T Felson

**Affiliations:** 1 Clinical Epidemiology Research and Training Unit, Boston University School of Medicine, Boston, MA 02118, USA; 2 Department of Rheumatology, Lapeyronie Hospital, Montpellier I University, UMR 5535 Montpellier, France; 3 Arthritis Research UK Epidemiology Unit, University of Manchester, Manchester, M13 9PT, UK

## Abstract

**Introduction:**

In development of the American College of Rheumatology (ACR)/European League Against Rheumatism (EULAR) rheumatoid arthritis (RA) remission definitions using clinical trials data, one criterion used to compare different remission definitions was whether, compared with those not in remission, those in remission had evidence of later disease stability defined by x-ray and functional status. Validation of the RA remission criteria using observational study data is necessary before recommending their use in practice.

**Methods:**

Using data from those who met RA criteria in the ESPOIR cohort, we matched each person in remission with a person not in remission and then carried out analyses comparing later stability of x-ray and health assessment questionnaire (HAQ) between the two groups. We compared the predictive validity of the same candidate definitions of remission evaluated in the ACR/EULAR process. To minimize potential bias and produce more stable results, we used a bootstrap resampling approach to select those not in remission, repeating the sample matching analysis process 500 times.

**Results:**

Results were similar to those of clinical trials analyzed for the ACR/EULAR remission criteria. Specifically, the ACR/EULAR remission definitions using either an simple disease activity index (SDAI) ≤ 3.3, clinical disease activity index (CDAI) ≤ 2.8 or a definition of remission requiring tender joint count, swollen joint count, patient global assessment all ≤ 1 performed as well or better than other candidate definitions of remission in terms of predicting later x-ray and function stability.

**Conclusions:**

ACR/EULAR definitions of remission developed for trials are similarly valid in observational studies in RA and could be used in practice.

## Introduction

Recently a committee consisting of members of the American College of Rheumatology (ACR) and the European League Against Rheumatism (EULAR) with help from Outcome Measures in Rheumatoid Arthritis Clinical Trials (OMERACT) produced a new rheumatoid arthritis (RA) remission definition for use in clinical trials [1]. While the committee developed a remission definition using only trial data, suggestions were made for remission definitions to be used in practice. The performance of the trial-based definitions in practice-based settings and of those proposed for use in practice has not been fully examined. If these new RA remission definitions perform as comparably in observational studies as in randomized clinical trials, then these might also be used as practice-based criteria.

While studies have examined the performance of these criteria in practice-based observational studies [2], one of the central elements of validating the criteria was to test their predictive validity, that is, whether being in remission at one time predicted good outcomes later. To our knowledge, there has been no attempt to corroborate the ACR/EULAR committee's examination of the predictive validity of different remission definitions in practice-based studies. Evidence from such studies could validate the use of the committee's recommendations in practice.

In this paper, we apply a matched bootstrap re-sampling method to an observational study, the French ESPOIR study, to validate the performance of the provisional ACR/EULAR RA remission criteria for use in practice.

## Materials and methods

### The ESPOIR cohort

The ESPOIR cohort [3,4] is a prospective observational study of adults aged 18 to 70 years recruited from 14 regions across France under the auspices of the French Society of Rheumatology, and with a protocol approved by the Montpelier University ethical committee. To be included, patients had to present with inflammatory arthritis lasting for 6 weeks to 6 months, involving more than two joints and diagnosed by the referring physician as RA or RA-like (that is, a high suspicion of RA). Patients had never previously undergone treatment with a disease-modifying anti-rheumatic drug (DMARD) or steroids. Patients were excluded if the referring physician judged they had other clearly defined rheumatic diseases.

Patients were recruited from general practitioners (GPs) and rheumatologists. Data were collected by the regional university rheumatology department which did not interfere with patient treatment. Patients were routinely treated and followed up by private rheumatologists in the geographical area, and in exceptional cases, by GPs with a special interest in rheumatology. All patients were followed by the same investigator every 6 months during the first two years and every year thereafter. Data on medical history, socio-economic and demographic characteristics, clinical, biological, radiographic and genetic parameters were also collected. Baseline and one-year radiographs of hands, wrists and feet were read according to the van der Heijde Sharp score, blinded to patient identity, patient characteristics and treatment, but with known time order for reasons of sensitivity to change [5]. The main radiographic results were recently published [4]. The first patients were enrolled in December 2002, and in total 813 patients were included, of whom 641 met the 2010 ACR/EULAR classification criteria for RA. This latter group is the focus of this report.

### Definition of remission

In the ACR/EULAR RA definition of remission for clinical trials, remission was defined based on whether a patient was in remission 6 months after they were randomized to an RA treatment, and good outcomes (after remission) were examined 12 to 24 months after randomization.

For this analysis, we defined the remission group as those who ever reached the ACR/EULAR RA remission criteria during the study, and defined the time to remission as the time they first reached remission. For those who were already in remission when they entered the study, time to remission was set to zero.

Those who never reached remission during the study were defined as the non-remission group.

To avoid the problem of making an arbitrary choice of a fixed time window to non-remission, we used a matched bootstrap re-sampling method to determine the time to non-remission from the non-remission group, so that potential bias and random variation were averaged out. Without re-sampling, we would choose one random time point for each person not in remission to match to a person in remission. With bootstrap re-sampling, we could create a more robust, precise and valid sampling of the range of values for persons not in remission. By the Law of Large Numbers, the sample mean approaches the population mean when sample size gets large, whereas a single observation is not a precise or especially valid estimator of the population mean.

We used Effron's Boostrap method [6,7] which allows one to estimate sample distributions of parameter estimates, and quantify uncertainty by calculating standard errors and confidence intervals of the parameter estimates so that statistical inference can be made. The basic idea of the bootstrap method is as follows: Step 1: resample; create a bootstrap sample with the same size as the original sample by resampling randomly with replacement from the original data. Step 2: perform the necessary data analysis using the bootstrap sample as if it were the original sample, and obtain parameter estimates of interest. Step 3: repeat Step 1 and Step 2 many times (depending on the nature of parameter estimation) and obtain the distribution of the parameter estimate. Step 4: construct standard errors and confidence intervals for parameters of interest.

### Application to the ESPOIR study for validation of ACR/EULAR RA remission criteria

We applied the bootstrap sampling approach to our evaluation of the predictive validity of different ways of defining remission in RA. We carried out analyses for all patients in the ESPOIR study starting at the baseline examination, and also in the subset of patients meeting ACR/EULAR criteria for RA at the baseline ESPOIR examination. We defined the remission group as persons who ever reached RA remission during the study and defined time to remission as the first time after entry that they reached remission according to successive 6-month windows, and then the proportions with remission within each 6-month window were calculated. Then bootstrap samples were selected for the non-remission group according to the same proportion from the remission group (for example, if 25% of the patients reaching remission reached it during the first 6-month window, we would choose 25% of the non-remission group in this window also.)

#### Definition of outcome

As with the ACR/EULAR committee's approach to defining remission [1], we examined predictive validity, defining it based on good radiographic and functional (health assessment questionnaire, HAQ) outcomes. For the ESPOIR cohort, we examined good outcomes over the interval of 12 to 24 months after the patient first reached remission.

We defined a good outcome for radiographic damage and physical function separately, and then characterized patients who had good outcomes on both measures. The good radiographic outcome was defined as stable x-ray diagnosis over one year (12 to 24 months after achieving remission) (change in total SHARP or van der Heijde modified total SHARP scores ≤ 0). A good functional outcome was defined as stable HAQ assessment [8] and maintenance of a low HAQ (HAQ change ≤ 0 and HAQ ≤ 0.5 during the year 12 to 24 months after achieving remission).

Positive likelihood ratios and their confidence intervals were computed using. a standard positive ratio calculator [9]. Likelihood ratios were used to compare the proportion of patients with RA in remission who had a good outcome, to the proportion of patients with RA not in remission who had a good outcome. To rank candidate definitions of remission, we used the *P-*value from the logistic regression chi-square test.

## Results

Table 1 shows the characteristics of patients in the ESPOIR study at the baseline examination. Patients tended to be female and to have active disease as evidenced by the presence of multiple tender and swollen joints.

**Table 1:** ESPOIR study demographics and disease characteristics for patients meeting ACR/EULAR classification criteria for rheumatoid arthritis at entry.

Variables	
Number	641

Age, years	48.5 (12.2)

Sex, number (%) female	499 (77.8)

Tender joint count, score from 0 to 28	9.8 (7.2)

Swollen joint count, score from 0 to 28	8.2 (5.5)

CRP, mg/dL	2.1(3.3)

Patient global assessment, cm from 0 to 10	6.2 (2.5)

Physician Global Assessment in cm from 0 to10	5.4 (2.2)

Pain score in cm from 0 to 10	5.7 (2.6)

HAQ, score from 0 to 3	1 (0.7)

Modified total Sharp score	6.4 (10.5)

IgM rheumatoid factor positivity, n (%)	367 (57.2)

Anti-CCP positivity, n (%)	313 (48.8)

Results presented as mean (SD) unless stated otherwise. Anti-CCP: test positive for antibody to cyclic citullinated protein

We examined the members of the ESPOIR cohort who met the criteria for RA [10] to evaluate the predictive validity of different definitions of remission as tested by the ACR/EULAR committee. Table 2 shows that the two new RA remission definitions (28 tender joint count (TJC28), 28 swollen joint count (SJC28), C-reactive protein (CRP) and patient global assessment (PtGA) ≤ 1, and simple disease activity index (SDAI) ≤ 3.3) performed among the best of the candidate remission definitions in predicting good outcomes for radiographic and HAQ stability during the one-year follow-up (in both the positive likelihood ratio and *P*-value).

**Table 2:** Comparison of the ACR/EULAR RA remission definition candidate for patients meeting ACR/EULAR classification criteria for rheumatoid arthritis (ESPOIR STUDY).

	Prevalence of good outcome in patients**:			
Candidate remission definition (* definitions are those recommended by ACR/EULAR)	**In remission**	Not in remission *	Positive likelihood ratio *(95% CI)	***P*-value**

**Boolean-based definition**				

TJC28, SJC28, CRP ≤ 1	25% (29/118)	12%	1.4 (1.1, 1.7)	0.0526

TJC28, SJC28, CRP, PhGA ≤ 1	32% (23/71)	13%	1.9 (1.4, 2.7)	0.0034

TJC28, SJC28, CRP, PtGA ≤ 1*	**39% (23/59)**	**12%**	**2.4 (1.6,3.5)**	**0.0002**

TJC28, SJC28, CRP, pain ≤ 1	34% (24/70)	13%	2.0 (1.4, 2.8)	0.0023

TJC28, SJC28, CRP, PhGA, PtGA ≤ 1	40% (22/55)	12%	2.6 (1.7, 3.8)	0.0002

TJC28, SJC28, CRP, PhGA, pain ≤ 1	38% (21/55)	13%	2.3 (1.6, 3.5)	0.0006

TJC28, SJC28, CRP, PtGA, pain ≤ 1	40% (21/53)	12%	2.6 (1.7, 3.9)	0.0002

TJC28, SJC28, CRP, PhGA, PtGA, pain ≤ 1	39% (20/51)	13%	2.5 (1.6, 3.9)	0.0005

**Index-based definition**				

DAS28 < 2.6	25% (35/146)	11%	1.3 (1.1, 1.5)	0.0383

DAS28 < 2.0	28% (24/85)	12%	1.6 (1.2, 2.2)	0.0148

SDAI ≤ 3.3*	**39% (27/69)**	**12%**	**2.3 (1.6, 3.2)**	**0.0001**

**Definitions without CRP (for clinical practice)**				

TJC28, SJC28, PhGA, PtGA ≤ 1	38% (23/60)	13%	2.3(1.6,3.4)	0.0003

TJC28, SJC28, PtGA < 1*	**37% (24/65)**	**12%**	**2.0(1.4,2.9)**	**0.0004**

CDAI ≤ 2.8*	**36% (26/72)**	**12%**	**2.0(1.5,2.8)**	**0.0005**

*Average values calculated from the 500 bootstrap samples of patients not in remission.

**Good outcome defined in a person when they had both good function and good radiographic outcomes (see Methods). CDAI: clinical disease activity index; CRP: C-reactive protein; DAS: disease activity score; EULAR: European League Against Rheumatism; PtGA: patient global assessment; PhGA: physician global assessment; RA: rheumatoid arthritis; SJC28: 28 swollen joint count; TJC28: 28 tender joint count.

We also confirmed that the ACR/EULAR committee recommendations for defining remission in clinical practice (TJC28, SJC28 and PtGA ≤ 1, and CDAI ≤ 2.8) worked well, in that those who reached remission had a higher chance of good future outcomes than those not reaching remission.

## Discussion

An evaluation of the predictive validity of different candidate definitions of remission in the ESPOIR cohort, a practice-based observational study, shows that the new ACR/EULAR trial-based remission definitions have high predictive validity for good outcomes in clinical practice.

Practice-based definitions suggested by the committee focused on those definitions that did not include acute phase reactants, which were felt to be difficult to obtain during a clinic visit. Our analyses validated the committee's choices and suggested that those recommended would perform well in practice. While the definitions recommended by the committee did not necessarily have the highest positive predictive values and lowest *P*-values of all those tested, they performed well, and using these definitions, the proportion in remission doing well was subsequently within 2% of the top-performing definitions (see Table 2).

Predictive validity was a critical element in the selection of definitions of remission for RA. It was felt by the ACR/EULAR committee that persons in remission at one time point should have more favorable later RA outcomes than persons not in remission. Our data suggest that the RA patients who attain the recommended definitions of remission in practice have a high likelihood of good future outcomes. As in the trial data analysis, our results suggest that the Boolean and SDAI/CDAI definitions of remission are better at predicting good outcomes than disease activity score (DAS)-based definitions of remission.

One other study has examined the relationship between different definitions of RA remission and functional and radiographic status [11]. While also focused on patients with recent onset disease, this used data from a trial, the BeSt study, which was not conducted in a practice based setting. In this study, versions of the DAS were as strongly associated with good outcomes as the ACR/EULAR-recommended definitions. It should be noted that the design of the BeSt study, in which patient treatment was guided by the DAS score [12] dictates that outcomes cannot be independent of the DAS score, and this makes it likely that DAS scores would be associated with major outcomes. Thus, it is not surprising that in this analysis of BeSt being in DAS remission portended good future outcomes.

Evaluating predictors of outcomes in observational studies like ESPOIR is not as straightforward as doing these analyses in clinical trials. First, it is hard to define a fixed time of remission. Among patients in some cohorts (although not in ESPOIR) we do not know when RA treatment was initiated; the majority might have already received multiple RA treatments before they enter the study. Second, some patients might already be in remission when they enter the study. Since the treatment protocol is not controlled as in a trial, it does not make sense to set a fixed time point after cohort entry as the time of remission, because some patients will reach remission at other time points. Also, because patient population heterogeneity is higher in observational studies than in clinical trials, some patients may respond more slowly or more quickly to new treatments than those in a trial.

There are also challenges in choosing a time point for "non-remission" so that valid comparisons can be made between non-remission and remission groups. The time to a non-event, that is, time to non-remission for a patient in an observational study, is either impossible to define or can only be arbitrarily defined. For those never reaching remission, it can be any time from baseline to the end of the study. Arbitrary fixing of the time of non-remission may introduce bias. Besides, since the time of remission for patients achieving remission is dynamic, it makes sense not to fix the non-remission time. We used bootstrap methods to create samples of non-remission patients. The well established advantages of bootstrap methods are that 1) they require fewer assumptions (for example, normality of the parameter estimates is not required); 2) they produce more precise and stable (that is, valid) parameter estimates than classical methods; 3) standard errors, confidence intervals and other parameters are easy to derive based on the distribution of the bootstrap parameter estimates to make inferences; and 4) the results are stable [6,7].

There are a number of limitations to our study. First, we limited our comparison of candidate definitions of remission to those evaluating predictive validity. Other considerations are important too, such as face and content validity, feasibility, and reproducibility. The ACR/EULAR committee considered some but not all of these in its deliberations. Remission was defined by the ACR/EULAR committee using a data-driven consensus process. This type of process used for all consensus efforts in rheumatology combines expert opinion and data analysis. Prior to data analysis, there was input from the committee (that included experts in RA research), as to which measures should be included in a remission definition; the committee dictated that swollen and tender joint counts and CRP were mandatory. Although we added other variables to these, this committee decision determined subsequent variable selection and heavily influenced the selection of candidate remission definitions. This could be regarded a controversial; other recommendations or even an agnostic variable-driven approach might have produced a different definition of remission and our analyses in this paper might also have tested other options as definitions, ones not considered by the ACR/EULAR committee.

Among the limitations of the study is that we studied only one observational cohort. More observational studies from different geographical regions and study populations are needed to make sure that our single validation is generalizable to other samples of patients. However, the data from in this study are from a comprehensive large nationwide cohort of persons with RA.

## Conclusions

The ACR/EULAR definitions of remission were developed using data from trials. We examined the predictive validity of these definitions of remission in the ESPOIR cohort, a practice-based observational cohort from France. We found that persons in ACR/EULAR remission in ESPOIR had a high rate of later radiographic and functional stability, and suggest that these definitions of remission are valid in clinical practice settings.

## Abbreviations

ACR: American College of Rheumatology; CDAI: clinical disease activity index; CRP: C-reactive protein; DAS: disease activity score; DMARD: disease modifying anti-rheumatic drugs; EULAR: European League Against Rheumatism; HAQ: health assessment questionnaire; OMERACT: Outcome Measures in Rheumatoid Arthritis Clinical Trials; PtGA: patient global assessment; RA: rheumatoid arthritis; SDAI: simple disease activity index; SJC28: 28 swollen joint count; TJC28: 28 tender joint count.

## Competing interests

The authors declare that they have no competing interests.

## Authors' contributions

BZ oversaw the analysis, conceptualized the project and drafted the manuscript. BC provided the data and revised the manuscript. NR carried out the analysis of the ESPOIR cohort and revised the manuscript. DTF conceptualized the project, directed the team and revised the manuscript. All authors have read and approved the final manuscript.
